# Supplementary material for: Accommodating heterogeneous missing data patterns for prostate cancer risk prediction
Source: BMC Med Res Methodol. 2022 Jul 21;22:200. doi: 10.1186/s12874-022-01674-x (PMC9306143; doi:10.1186/s12874-022-01674-x)

**Supporting information**

**Table S1.** Algorithms for the 6 risk modeling approaches. Starting variables are available risk factors from the user. Used and cohort variables are subsets or all of the starting variables corresponding to those used by the model and those with less than 40% missing rates in the cohort, respectively.

| **Algorithm: Available cases** | | |
| --- | --- | --- |
| 1 | Subset the PBCG dataset by records without missing values for ‘starting variables’ | |
| 2 | Fit main effects logistic regression model with ‘starting variables’ to the PBCG subset pooled for all cohorts | |
| **Algorithm: Iterative BIC selection** | | |
| 1 | used variables = starting variables | |
| 2 | while number of ‘used variables’ reduces do | |
| 3 |  | Subset the PBCG dataset (pooled for all cohorts) by ‘used variables’ |
| 4 |  | Use only complete records |
| 5 |  | Perform logistic regression BIC selection starting with main effects up to two-way interactions |
| 6 |  | used variables = variables in the selected model (either as main effect or as interaction) |
| 7 |  | risk = Predict clinically significant prostate cancer risk with information in ‘starting variables’ |
| 8 | end while | |
| **Algorithm: Cohort ensemble** | | |
| 1 | cohort variables = variables of each cohort with less than 40% missing records | |
| 2 | for all cohorts do | |
| 3 |  | used variables = variables of ‘starting variables’ that are in ‘cohort variables’ |
| 4 |  | Subset the PBCG dataset by cohort, and ‘used variables’ |
| 5 |  | Use only complete records |
| 6 |  | Perform logistic regression BIC selection starting with main effects up to two-way interactions |
| 7 |  | used variables[cohort] = variables in the selected model (either as main effect or as interaction) |
| 8 |  | risk[cohort] = Predict clinically significant prostate cancer risk with information in ‘starting variables’ |
| 9 | end for | |
| 10 | overall risk = mean(risk) | |
| **Algorithm: Categorization** | | |
| 1 | Categorize all predictor variables with the additional factor not available (NA). Continuous variables with missing values are categorized. | |
| 2 | Fit main effects logistic regression model with all variables to the PBCG dataset pooled for all cohorts | |

| **Algorithm: Missing indicator** | | | |
| --- | --- | --- | --- |
| 1 | | Categorize all categorical predictor variables with the additional factor missing. Add for continuous variables with missing values an indicator variable whether the variable was missing or not | |
| 2 | | Fit main effects logistic regression model with all variables and include the interactions of the indicator with the corresponding variable in the model to the PBCG dataset pooled for all cohorts | |
| **Algorithm: Imputation** | | | |
| 1 | Run 30 imputations by chained equations (mice) on the training PBCG dataset | | |
| 2 | for all imputed datasets do | | |
| 3 |  | | Fit main effects logistic regression model with all variables in the model to the imputed PBCG dataset pooled for all cohorts |
| 4 | end for | | |
| 5 | Impute missing values in the test case with means of the training set | | |
| 6 | Average the coefficients from mice for use in the prediction models | | |

**Figure S1**. Differences between the cohorts in terms of distributions of the twelve risk factors and their associations with clinically significant prostate cancer. NA = missing.


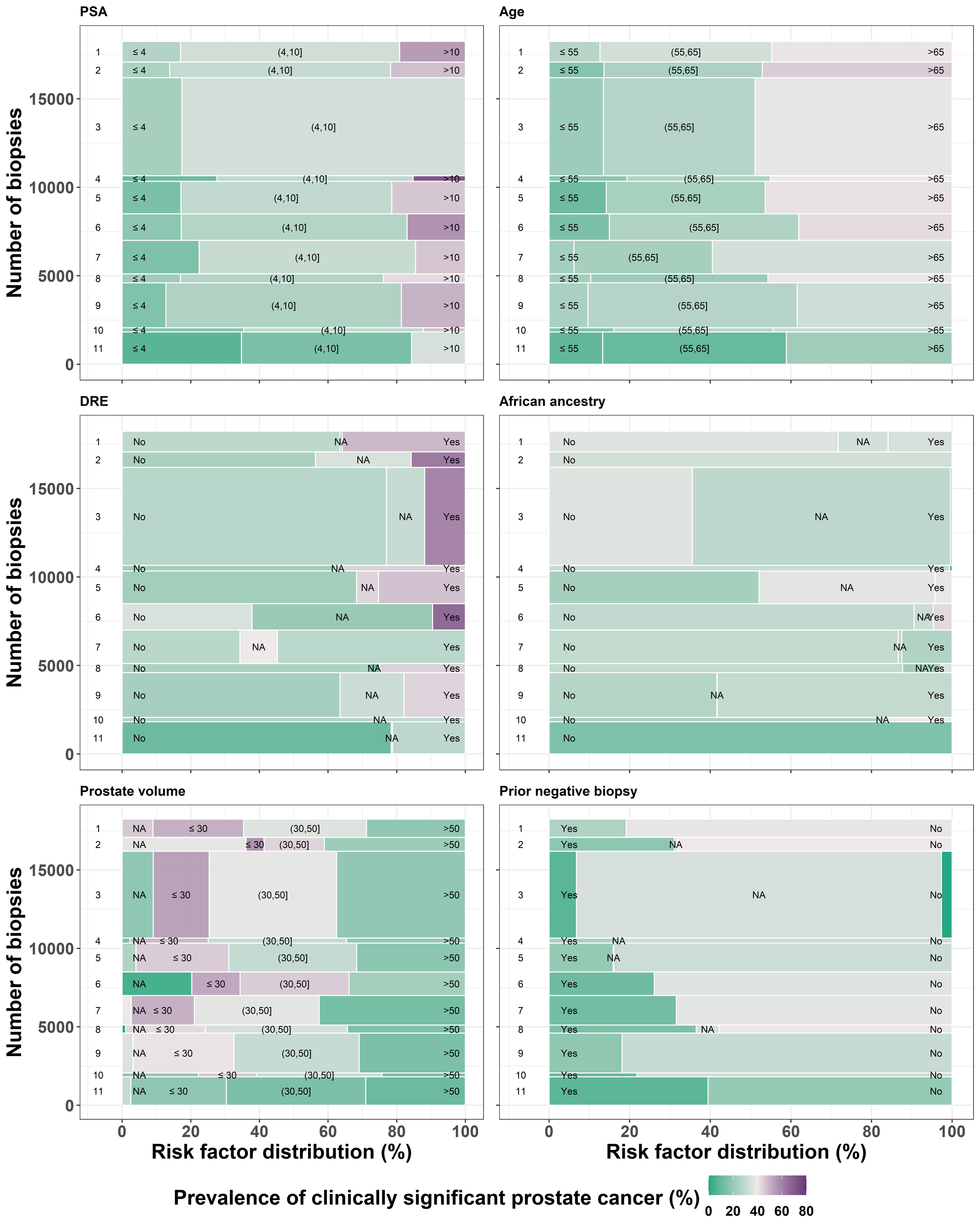


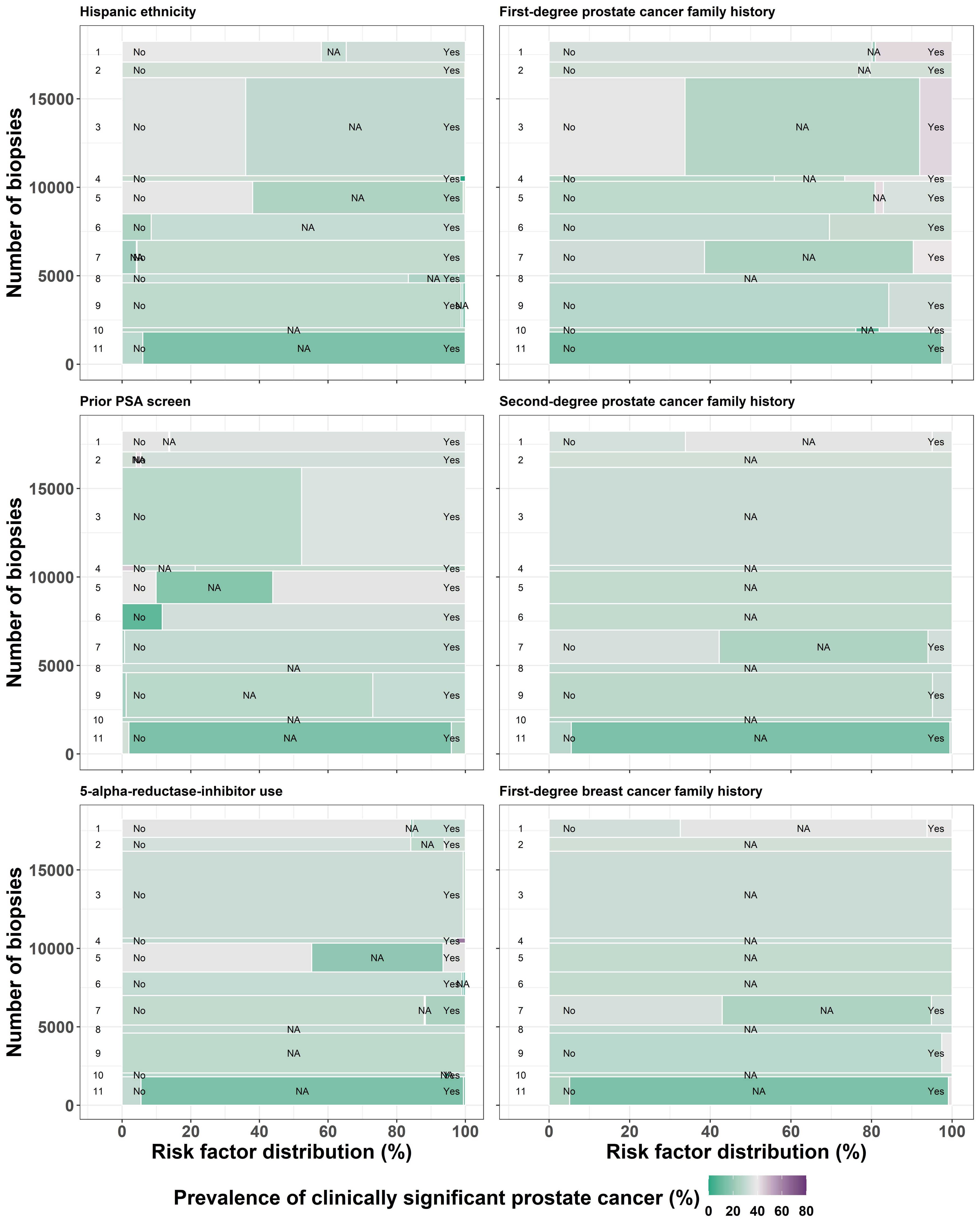

Supplement: Supplementary file 1 — Additional file 1: Detailed description of the algorithms for the 6 risk modeling approaches. Differences between the cohorts in terms of distributions of the twelve risk factors and their associations with clinically significant prostate cancer. [file 12874_2022_1674_MOESM1_ESM.docx]
